# A synthetic retinoic acid receptor agonist Am80 ameliorates renal fibrosis via inducing the production of alpha-1-acid glycoprotein

**DOI:** 10.1038/s41598-020-68337-z

**Published:** 2020-07-10

**Authors:** Hiroshi Watanabe, Jing Bi, Ryota Murata, Rui Fujimura, Kento Nishida, Tadashi Imafuku, Yuka Nakamura, Hitoshi Maeda, Ayumi Mukunoki, Toru Takeo, Naomi Nakagata, Yuki Kurauchi, Hiroshi Katsuki, Motoko Tanaka, Kazutaka Matsushita, Masafumi Fukagawa, Toru Maruyama

**Affiliations:** 1 Department of Biopharmaceutics, Graduate School of Pharmaceutical Sciences, Kumamoto University, 5-1 Oe-honmachi, Chuo-ku, Kumamoto, 862-0973 Japan; 2 Program for Leading Graduate Schools “HIGO (Health Life Science: Interdisciplinary and Global Oriented) Program”, Kumamoto University, Kumamoto, Japan; 3 Division of Reproductive Engineering, Center for Animal Resources and Development (CARD), Kumamoto University, Kumamoto, Japan; 4 Department of Chemico-Pharmacological Sciences, Graduate School of Pharmaceutical Sciences, Kumamoto University, Kumamoto, Japan; 5 Department of Nephrology, Akebono Clinic, Kumamoto, Japan; 6 Division of Nephrology, Endocrinology and Metabolism, Tokai University School of Medicine, Kanagawa, Japan

**Keywords:** Renal fibrosis, Chronic inflammation

## Abstract

Renal fibrosis is a major factor in the progression of chronic kidney disease and the final common pathway of kidney injury. Therefore, the effective therapies against renal fibrosis are urgently needed. The objective of this study was to investigate the effect of Am80, a synthetic retinoic acid receptor (RAR) agonist, in the treatment of renal interstitial fibrosis using unilateral ureteral obstruction (UUO) mice. The findings indicate that Am80 treatment suppressed renal fibrosis and inflammation to the same degree as the naturally-occuring retinoic acid, all-trans retinoic acid (atRA). But the adverse effect of body weight loss in Am80-treated mice was lower compared to the atRA treatment. The hepatic mRNA levels of alpha-1-acid glycoprotein (AGP), a downstream molecule of RAR agonist, was increased following administration of Am80 to healthy mice. In addition, increased AGP mRNA expression was also observed in HepG2 cells and THP-1-derived macrophages that had been treated with Am80. AGP-knockout mice exacerbated renal fibrosis, inflammation and macrophage infiltration in UUO mice, indicating endogenous AGP played an anti-fibrotic and anti-inflammatory role during the development of renal fibrosis. We also found that no anti-fibrotic effect of Am80 was observed in UUO-treated AGP-knockout mice whereas atRA treatment tended to show a partial anti-fibrotic effect. These collective findings suggest that Am80 protects against renal fibrosis via being involved in AGP function.

## Introduction

Approximately 10% of the world’s population are affected by chronic kidney disease (CKD). CKD is associated with a risk of end-stage renal disease (ESRD), which is fatal without kidney replacement therapy such as dialysis or kidney transplantation^1,2^. Renal fibrosis is the main factor in CKD progression and the final common pathway of kidney injury^3,4^. Although initial fibrosis is thought to play a beneficial role in maintaining the kidney structure upon injury and repair^5^, the development of fibrosis causes a progressive decline in renal function^6^. Therefore, effective therapies against renal fibrosis are urgently needed.

It is known that retinoids, derivatives of vitamin A (retinol), regulate embryonic development and cellular differentiation^7^. All-trans retinoic acid (atRA) is a natural retinoic acid that is clinically used for treating patients with acute promyelocytic leukemia. Retinoids are also of interest for their renoprotective and anti-inflammatory effects. In fact, atRA treatment was reported to suppress renal fibrosis in a unilateral ureteral obstruction (UUO)-induced renal fibrosis model^8^, and retinoids have protective effects in numerous types of renal damage and inflammation including acute pyelonephritis^9^ and glomerulosclerosis in adriamycin-induced nephropathy model^10,11^ In addition, Chiba et al*.* reported that atRA treatment reduced macrophage-dependent injury and fibrosis after an acute kidney injury (AKI), where atRA functioned to regulate macrophage activation^12^. These collective studies indicate that retinoids play a protective role in renal injury. However, the detailed mechanism of how retinoids protect against renal fibrosis is not fully understood.

To improve the medicinal properties of natural retinoic acid atRA, Am80, or tamibarotene, was developed for use as a synthetic retinoid^13^. While atRA binds to the retinoic acid receptor (RAR)-α, RAR-β and RAR-γ, Am80 binds only to RAR-α and RAR-β. According to previous research, Am80 is approximately 10 times more potent than atRA as an in vitro inducer of differentiation in the human leukemia cell lines NB-4 and HL-60^14^. Moreover, compared with atRA, the plasma concentration of Am80 in healthy volunteers can be maintained for a longer time, suggesting that the plasma clearance of Am80 is rather low^14,15^. Considering the above factors, in terms of therapeutic effect, Am80 would be expected to be clinically superior to atRA^15,16^. However, the issue of whether or not Am80 protects against renal fibrosis remains uninvestigated.

Alpha-1-acid glycoprotein (AGP), also known as orosomucoid (ORM), is a major acute-phase protein. There are two subtypes of AGP genes in humans, *ORM1*, which codes for the F1*S variant, and *ORM2*, which codes for the A variant. In mice there are three subtypes of AGP genes, *Orm1*, *Orm2* and *Orm3.* The plasma concentration of AGP is 0.5 g/L, and this level is increased by 2–5 times following the onset of an acute-phase response. AGP also has renoprotective effects. It has been reported that exogenously administered AGP exerts protective effects against several types of renal damage including aminonucleoside-induced minimal change nephrosis^17^, puromycin-induced renal injury^18^ and ischemia/reperfusion injury^19^. In our recent studies, we found that AGP is protective against obstructive nephropathy and renal fibrosis^20^. AGP is also known for its anti-inflammatory actions. In a previous study, we demonstrated that AGP inhibited the production of IL-6 and TNF-α and induced the expression of CD163, a specific marker that has anti-inflammatory potential and is expressed predominantly on monocytes/macrophages^21^. In addition, Nakamura et al*.* proposed that AGP stimulates monocytes to polarize M2b monocytes^22^. Importantly, Mouthiers et al*.* reported that retinoids cause an increase in AGP expression at the transcriptional level in rat hepatocytes^23^. Taken together, it therefore appears that AGP may act as a downstream molecule in the anti-renal fibrotic effect of retinoids as RAR agonists.

The objective of this research was to compare the effects of Am80 versus atRA, to elucidate the function of endogenous AGP and to explore the relationship between AGP and retinoids, especially Am80 and atRA, using a UUO-induced renal fibrosis model.

## Results

### Am80 suppresses renal fibrosis and inflammation similar to atRA in UUO-mice

**Figure 1:**
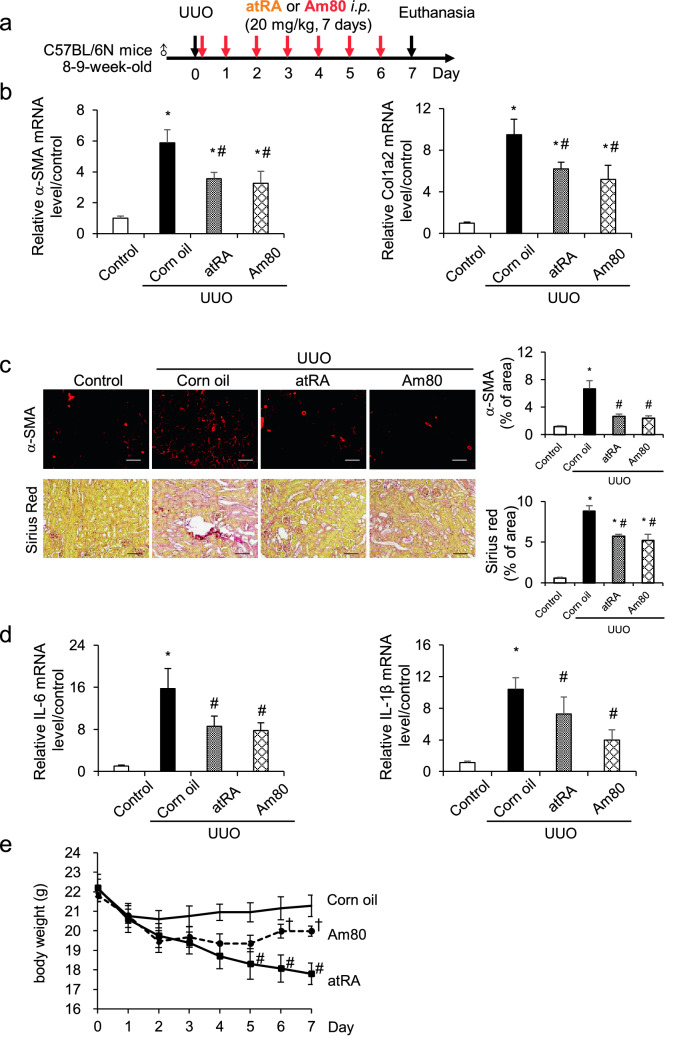
Am80 suppresses renal fibrosis and inflammation in UUO mice at a level similar to that for atRA. (**a**) atRA or Am80 were administered i.p. in corn oil at a dose of 20 mg/kg, each day (from day 0 to day 6) after the UUO treatment. (**b**) Treatment of Am80 or atRA reduced the mRNA expression of α-SMA and Col1a2 at day 7. (**c**) a-SMA was immunostained and the accumulation of collagen in kidney was detected by picrosirius red staining. Scale bar, 100 μm. (**d**) Treatment of Am80 or atRA reduced the mRNA expression of IL-6 and IL-1β at day 7 (**e**) The side effect of body weight loss was observed in atRA-treated UUO mice, but not in the Am80 treated UUO mice. *P < 0.05 compared with control; ^#^P < 0.05 compared with corn oil; ^†^P < 0.05 compared with atRA (n = 6 for control group; n = 10 for each UUO group). Data are presented as the mean ± SE.

We compared the anti-fibrotic effects of Am80 with atRA in a UUO-induced renal fibrosis model. Male C57BL/6N mice (8–9 weeks of age, Japan SLC) were subjected to UUO treatment following a previously reported method^20^. The mice were administered Am80 and atRA immediately after the UUO treatment for 7 days, *i.p.*, at a once daily dose of 20 mg/kg (Fig. 1a). Kidney samples were harvested 7 days after the UUO treatment. The results for qRT-PCR showed that the mRNA expression of α-SMA and Col1a2 were increased in UUO mice that had received the corn oil treatment, and these mRNA levels were significantly suppressed to the same degree in mice that had been treated with either Am80 or atRA (Fig. 1b). Immunostaining of α-SMA and picrosirius red staining showed similar results, in that both Am80 or atRA treatments resulted in the suppression of fibrosis in the UUO-treated mice (Fig. 1c). These data suggest that Am80 plays important roles in preventing the development of renal fibrosis and that the effect of Am80 was similar to that of atRA. We further studied the effect of Am80 and atRA on renal inflammation in UUO mice. The qRT-PCR results showed that the expression of IL-6 and IL-1β mRNA were both increased in the UUO mice, and these expressions were significantly suppressed by treatment with either Am80 or atRA (Fig. 1d). These results suggest that Am80 exerted an anti-inflammatory effect in the UUO pathology, as was observed in the case of the atRA treatment.

The body weights of the UUO-treated mice are summarized in Fig. 1e. The body weights of the atRA-treated UUO mice were significantly lower compared with the values for the corn oil-treated or Am80-treated UUO mice. Interestingly, no significant body weight changes were observed between the corn oil-treated group and the Am80-treated group. These results suggest that Am80 exerted a less adverse effect on body weight loss compared with atRA.

### Am80 treatment increased AGP production both in vivo and in vitro

**Figure 2:**
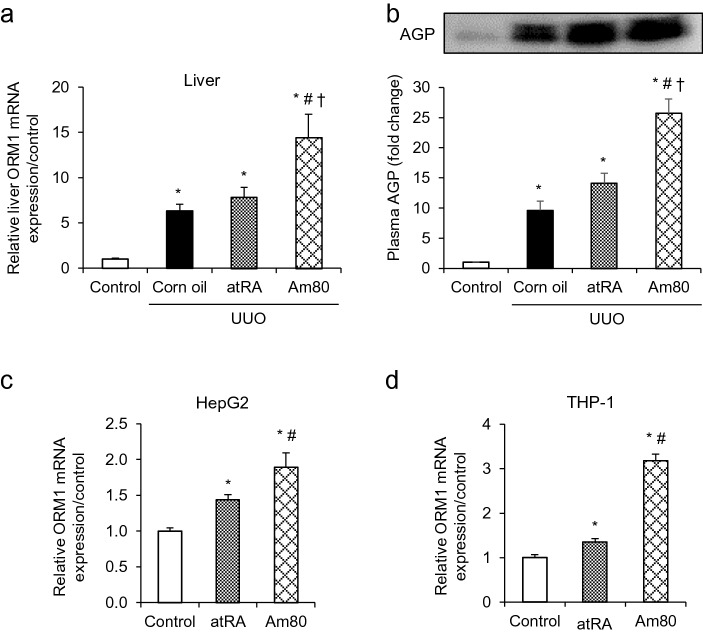
Am80 treatment increases ORM1 expression both in vivo and in vitro*.* (**a**) Hepatic ORM1 expression in the UUO-treated mice at day 7. (**b**) Plasma AGP protein expression in the UUO-treated mice at day 7. *P < 0.05 compared with the control mice. The full-length gel and bands are included in the Supplementary Fig. 2. ^#^P < 0.05 compared with the corn oil-treated UUO mice. ^†^P < 0.05 compared with the atRA-treated UUO mice. (n = 4 for control group; n = 5 for each UUO group) (**c**, **d**) mRNA expression of ORM1 in HepG2 and THP-1 cells after incubation with atRA or Am80 (0.1 μM). *P < 0.05 compared with control; ^#^P < 0.05 compared with atRA-treated group. Data are expressed as the mean ± SE (n = 3 for control; n = 4 for atRA or Am80 treatment).

Given that atRA activates the expression of the AGP gene (*ORM*) in rat hepatocytes via RAR-α and RXR-α^23^, and the fact that AGP functions as an anti-inflammatory protein, we hypothesized that AGP plays an important role as an anti-inflammatory agent as a RAR agonist. We first collected liver samples of the mice at 7 days after the UUO treatment to examine whether AGP is induced by retinoid in vivo. As a result, the mRNA expression of *ORM1* was increased in all of the UUO-treated mice. Specifically, hepatic ORM1 production in Am80-treated UUO mice were significantly enhanced compared with the other groups (Fig. 2a). Plasma AGP is mainly secreted from liver, so plasma AGP level was measured by western blots analysis. The data showed that the plasma AGP level was also increased by the administration of Am80 (Fig. 2b). This result confirms that the administration of Am80 led to a significant production of ORM1 in vivo condition.

AGP is produced mainly by the liver and is also produced by some extra-hepatic cells such as monocyte or macrophage^20^. Therefore, increased plasma AGP or increased AGP (*ORM1*) expression in renal macrophage could contribute to the anti- inflammatory effect in kidney. To examine whether AGP production can be induced by retinoids in vitro, we stimulated HepG2 hepatocytes with Am80 and atRA, respectively, at a concentration of 0.1 μM for 24 h. The results showed that the mRNA expression of *ORM1* was significantly increased in atRA-stimulated cells, and that the AGP-inducing activity of Am80 was stronger than that for atRA (Fig. 2c). In a similar manner, we stimulated THP-1-derived macrophages with Am80 and atRA, respectively, at a concentration of 0.1 μM for 48 h. Consistent with the results for HepG2 cells, the mRNA expression of *ORM1* was significantly increased in atRA-stimulated cells, and Am80 showed an even stronger *ORM1*-inducing activity than atRA in THP-1-derived macrophages (Fig. 2d). These collective results suggest that Am80 has stronger AGP (ORM1)-inducing activities compared with atRA, both in vivo and in vitro.

### Renal fibrosis and inflammation are exacerbated in AGP-KO mice after UUO treatment compared with WT mice

AGP-KO (mouse AGP genes: *Orm1*, *Orm2* and *Orm3* triple KO) mice with a C57BL/6N background were generated with the assistance of the Institute of Resource Development and Analysis Center for Animal Resources and Development (CARD), Kumamoto University, Japan. This is the first *Orm1-Orm2-Orm3* triple KO mice to have been developed. AGP genes (*Orm1*, *Orm2* and *Orm3*), plasma AGP protein and hepatic mRNA were not detected in the AGP-KO mice (Supplemental Fig. 1). No significant difference in appearance and growth was detected in the AGP-KO mice at 8 weeks of age, compared with the WT mice.

**Figure 3:**
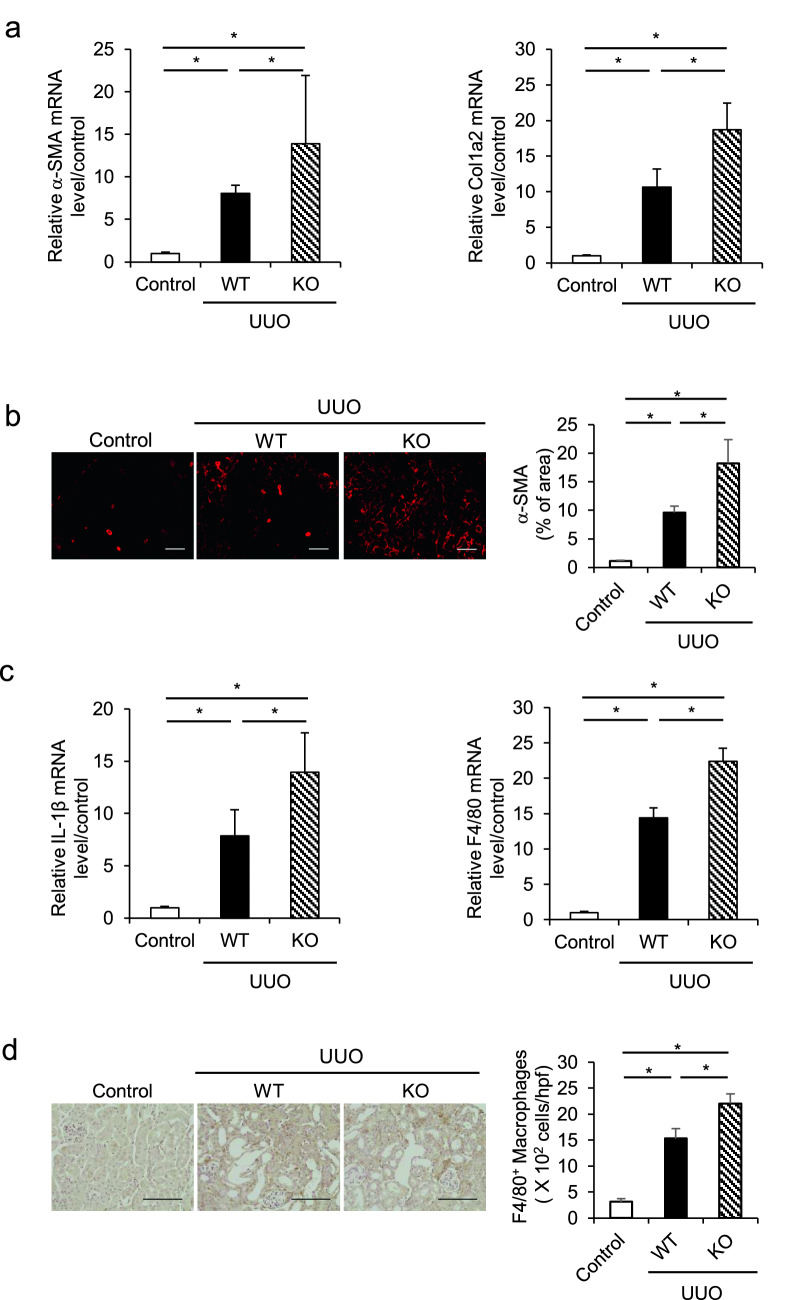
AGP-KO mice showed an exacerbated renal fibrosis and inflammation after the UUO treatment compared with WT mice. (**a**) C57BL/6N WT mice and AGP-KO mice were subjected to UUO treatment. The mRNA expression of α-SMA and Col1a2 was further increased in AGP-KO mice compared with WT mice. (**b**) Immunostaining of α-SMA in kidney was shown. (**c**) mRNA expression of IL-1β and F4/80 was exacerbated in AGP-KO mice compared with WT mice. (**d**) Immunohistochemistry of F4/80 in kidney was shown. *P < 0.05. Data are presented as mean ± SE (n = 4 for control group; n = 9 for each UUO group).

We evaluated the role of endogenous AGP in UUO-induced renal fibrosis. Male C57BL/6N WT and AGP-KO mice were subjected to UUO treatments and kidney samples were harvested at 7 days after the UUO treatment. The qRT-PCR results showed that the mRNA expression of α-SMA and Col1a2 were increased in WT mice, and these expressions were further increased in AGP-KO mice (Fig. 3a). Similar result was also observed in the immunostaining of α-SMA (Fig. 3b). These data suggest that endogenous AGP plays an important role in preventing the development of renal fibrosis.

We also examined the effect of an AGP-deficiency in renal inflammation. The qRT-PCR results showed that the mRNA expression of IL-1β in the kidney was further increased in the AGP-KO mice compared with WT mice. Similarly, we also found that F4/80 (a macrophage marker) in the kidney was increased in AGP-KO mice compared to WT mice (Fig. 3c and d). These results suggest that endogenous AGP suppressed the infiltration of macrophages, especially the pro-inflammatory subtype, into the kidney.

### Am80 failed to alleviate UUO-induced renal fibrosis in AGP-KO mice

**Figure 4:**
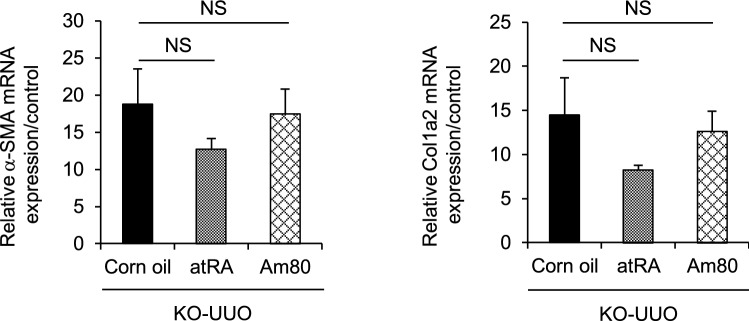
Am80 treatment fails to alleviate UUO-induced renal fibrosis in AGP-KO mice. The mRNA expression of α-SMA and Col1a2 in the UUO-treated AGP-KO mice were not significantly alleviated by Am80 administration (n = 4 for corn oil group; n = 5 for atRA or Am80 group).

To verify the hypothesis that a relationship exists between AGP and the anti-fibrotic and anti-inflammatory effect of RAR agonists, UUO-treated AGP-KO mice were administered Am80 and atRA 7 days, *i.p.*, at a dose of 20 mg/kg each day. Interestingly, the qRT-PCR results showed that the increased mRNA expression of α-SMA and Col1a2 in the UUO-treated AGP-KO mice were not significantly alleviated by Am80 administration whereas atRA administration tended to suppress (but not significantly) the expressions of α-SMA and Col1a2. These results confirm that the anti-fibrotic effect of Am80 is related to the induction of AGP (Fig. 4).

## Discussion

In this study, we compared the effects of Am80 with atRA on a UUO-induced renal fibrosis model, and confirmed that AGP has a role in the anti-fibrotic effects of these RAR agonists. We first found that Am80 showed similar anti-fibrotic and anti-inflammatory effects with atRA against UUO-induced renal fibrosis model. However, the adverse effect of body weight loss in Am80-treated mice was significantly lower than that mice that were treated with atRA. Secondly, using AGP-KO mice, we showed that endogenous AGP plays an anti-fibrotic and anti-inflammatory role during the development of UUO-induced renal fibrosis. Thirdly, we found that an anti-fibrotic effect of Am80, which was demonstrated in UUO-treated WT mice, was not observed in UUO-treated AGP-KO mice whereas the atRA treatment tended to show a partial anti-fibrotic effect in UUO-treated AGP-KO mice. The collective findings suggest that Am80 protects against UUO-induced renal fibrosis via the action of AGP.

Nakamura et al. demonstrated that, in order to fight against renal fibrosis, a wound healing process of fibroblast-to-myofibroblast occurs, and that myofibroblasts develop the ability to produce retinoic acid to support tubular regeneration^24^. Consequently, it would be reasonable to expect that Am80 would exert a renoprotective effect similar to that for atRA. As far as we know, the only research that examined the anti-fibrotic effect of Am80 was focused on bleomycin-induced dermal fibrosis. As reported by their findings, Am80 suppressed dermal fibrosis by modulating phenotypes of fibroblasts, endothelial cells and immune cells^25^. It was particularly noteworthy that Am80 ameliorated the pathologic events including the epithelial-mesenchymal transition (EMT), fibroblast activation and macrophage infiltration^25^. We can therefore confirm for the first time that Am80 is beneficial in suppressing renal fibrosis and inflammation caused by a UUO-treatment.

We recently demonstrated that exogenously administered AGP suppressed UUO-induced renal fibrosis and inflammation^20^. In vitro experiments using THP-1-derived macrophages also showed that an AGP treatment prevented lipopolysaccharide-induced macrophage activation^20^. In this study, to elucidate the role of endogenous AGP in a renal fibrosis model, we, for the first time, developed the *Orm1-Orm2-Orm3* triple knockout mice (AGP-KO mice). We compared the differences between WT mice and AGP-KO mice after a UUO-treatment. As a result, AGP-KO showed an increased expression of α-SMA, Col1a2 and IL-1β in the kidney compared with WT mice. These data suggest that endogenous AGP plays an anti-fibrotic and anti-inflammatory role during the development of UUO-induced renal fibrosis. Interestingly, F4/80, a macrophage marker, was also increased in the kidney of AGP-KO mice. The sustained activation of macrophages can lead to aberrant repair and the uncontrolled production of inflammatory cytokines, contributing to a state of persistent injury, which then leads to the progress of renal fibrosis^26^. In the present case, it is likely that the infiltration of inflammatory macrophages related to IL-1β expression could be suppressed by endogenous AGP in the pathological process. IL-1 β plays an important role in activating inflammasomes in a series of renal disease models, including diabetic nephropathy^27^ and UUO-induced renal fibrosis^28^. The sustained production of IL-1 β through NLRP3 inflammasome activation in macrophages has been shown to be a major driver of persistent inflammation and fibrosis in the liver^26,29^. In addition, Shimodaira et al*.* reported that under conditions of IL-1β stimulation, lung fibroblasts that were co-cultured with THP-1-derived macrophages produced an pro-fibrotic molecules such as osteopontin, separately, showing fibroblast/macrophage crosstalk that contributes to inflammation-associated fibrosis^30^. Consequently, therapeutic strategies that involve the IL-1β suppressive activity of AGP might contribute to regulating macrophage and inflammatory status.

RARs are regarded as potent regulators of inflammatory responses, and AGP is a protein that has anti-inflammatory and immune modulating activities. Although RAR agonists cause an increase in the expression of AGP at the transcriptional level, their relationship to their biological activity remains unexplored. Sierra-Mondragon et al. reported that atRA ameliorated the expression IL-6, which was mediated by TLR4/NF-κB during the initiation of diabetic nephropathy^31^. Another study demonstrated that the increased IL-6, IL-1β and MCP-1 mRNA levels were observed in a mouse model of experimental periodontal disease, and this was suppressed by an Am80 treatment^32^. Interestingly, when AGP was added to LPS-stimulated THP-1-derived macrophages, the mRNA expression of IL-6 and IL-1β were also significantly suppressed^20^. Consistent with these studies, we demonstrated that the Am80 treatment protected against UUO-induced renal fibrosis and inflammation, as evidenced by the suppression of IL-6 and IL-1β expression, while Am80 failed to show anti-fibrotic effects in AGP-KO mice. Considering that Am80 mediated the induction of AGP both in vivo and in vitro (Fig. 2), these findings serve to underscore the therapeutic potential of Am80-mediated AGP production in inflammatory diseases.

Based on our research findings, Am80 and atRA showed similar renoprotective effects in UUO-treated WT mice. It is noteworthy that the atRA treatment on UUO-treated AGP-KO mice tended to show anti-fibrotic effects to some extent while Am80 did not (Fig. 4). Two factors could be contributing to these results. First, Am80 has a higher AGP-inducing potential than atRA. In corn oil-treated UUO mice, the hepatic expression of *ORM1* mRNA showed a fivefold increase, while in Am80-treated UUO mice the increase was 15-fold (Fig. 2). At the same time, in atRA-treated UUO mice, no significant increase in hepatic *ORM1* production compared with the corn oil-treated UUO mice was found. This result indicates that the induction of AGP contributes to the anti-fibrotic effects of Am80. Secondly, while atRA binds to RAR-α, RAR-β and RAR-γ, Am80 has little affinity for RAR-γ. In the adult kidney, RAR-γ is expressed in proximal tubules while RAR-α and RAR-β are expressed in distal tubules^24^. It is possible that AGP exerts its anti-fibrotic effect in the downstream of RAR-α and RAR-β but not RAR-γ. In other words, it may be possible that RAR-γ mediates anti-fibrotic effects via an AGP-independent pathway. It would be also interesting to the further study in the signaling pathway and the location of RAR activation in renal fibrosis. Further research will clearly be needed to clarify this RAR-AGP axis.

In the present study, we found that the potency of Am80 for suppressing renal fibrosis and inflammation was comparable to that of atRA, but the side effects of body weight loss were avoided in the case of the Am80 treatment (Fig. 1). This result suggests that Am80 has the potential for being clinically advantageous in preventing severe adverse events including the differentiation syndrome. Furthermore, based on our findings, it is highly possible that Am80 exerts renoprotective effect via the function of AGP (Fig. 4). Based on our previous findings that in the protective effects of AGP against UUO-induced fibrosis, body weight loss was not observed in AGP-treated mice^20^, the fact that AGP could function as an anti-fibrotic therapeutic with less adverse effects makes its use promising.

## Conclusion

In this study, we found that Am80 exerts anti-fibrotic effects without body weight loss. In addition, endogenous AGP played an anti-fibrotic role during the development of renal fibrosis. Using AGP KO mice, we found that Am80 protects against renal fibrosis via the function of AGP. This study is the first evidence to show that AGP contributes to the anti-fibrotic and anti-inflammatory effects of RAR agonists. Thus, Am80-AGP pathway could serve as a potential target for the treatment of renal fibrosis.

## Materials and methods

### Animal model of unilateral ureteral obstruction (UUO)

Male C57BL/6 N WT mice (8 week-old, 23.0 ± 1.1 g (mean ± SD), Japan SLC) or AGP-KO mice (8 week-old, 22.6 ± 1.1 g (mean ± SD), CARD, Kumamoto University, Japan) were randomized by body weight. Treatment of UUO was previously described in detail^20^. After the UUO treatment, atRA (Wako, 20 mg/kg) or Am80 (Nippon Shinyaku, Kyoto, Japan, 20 mg/kg) was intraperitoneally administered each day. The UUO group was injected with an equivalent amount of corn oil. We sacrificed the mice at day 7 after surgery. All animal experiments were conducted with procedures approved by the experimental animal ethics committee at Kumamoto University and all methods were performed in accordance with the relevant guidelines and regulations.

### qRT-PCR

Isolation of total RNA from kidney or cells and measurement of qRT-PCR were performed as previously described^21^. Each primer sequence is shown in below (Supplemental Table 1).

### Immunostaining assay

Infiltration of macrophages was confirmed by immunostaining using F4/80 antibody (eBioscience, San Diego, CA). Immunofluorescence staining of kidney tissues for α-SMA was performed following the previously reports^20^. Secondary antibody Alexa Fluor 647 chicken anti-rabbit IgG (H + L) (1:200, Invitrogen, Carlsbad, CA) was used. The slides were observed using a BZ-X700 microscope (original magnification power × 200). The images were randomly acquired, with 8 to 10 high power fields collected for each mouse and then quantified within them.

### Picrosirius red staining

Accumulation of collagen in obstructed kidney was detected by picrosirius red staining^20^. After deparaffined and rehydrated, the sections (2 μm) were stained in picrosirius red for 1 h, and washed three times with 0.5% acetic acid solution. The slides were viewed with a BZ-X700 microscope (original magnification power × 400).

### Cell culture

The cell culture of HepG2 and THP-1 cells were carried out in accordance with previously reported method^20^. HepG2 cells were seeded at 2.0 × 10^5^ cells/well in 6-well plates and stimulated with atRA or Am80 at a concentration of 0.1 μM for 24 h. The phorbol 12-myristate 13-acetate-differentiated THP-1 cells were seeded at 1.0 × 10^6^ cells/well in 6-well plates and incubated with atRA or Am80 at a concentration of 0.1 μM for 48 h.

### Generation of AGP-KO mice

AGP-KO mice were efficiently generated by reproductive technology using ultrasuperovulation technique at the Institute of Resource Development and Analysis Center for Animal Resources and Development (CARD), Kumamoto University, Japan^33^. AGP KO (C57BL/6N-Orm2tm1(KOMP)Vlcg/Mmucd, RRID: MMRRC_048914-UCD) sperm was purchased from Knockout Mouse Project (KOMP) Repository, UC Davis, US.

### Statistical analysis

Data from animal and cell studies were compared by analysis of variance followed by Tukey’s multiple comparison. All results are expressed as the mean ± SE of the indicated experiments. A *P* value < 0.05 was considered to be statistically significant.

## Supplementary information

Supplementary information.
