# Supplementary material for: A synthetic retinoic acid receptor agonist Am80 ameliorates renal fibrosis via inducing the production of alpha-1-acid glycoprotein
Source: Sci Rep. 2020 Jul 10;10:11424. doi: 10.1038/s41598-020-68337-z (PMC7351735; doi:10.1038/s41598-020-68337-z)
Supplement: Supplementary file 1 — Supplementary information. [file 41598_2020_68337_MOESM1_ESM.docx]

**A synthetic retinoic acid receptor agonist Am80 ameliorates renal fibrosis *via* inducing the production of alpha-1-acid glycoprotein**

Hiroshi Watanabe^1,*^, Jing Bi^1,2,*^, Ryota Murata^1,*^, Rui Fujimura^1,2^, Kento Nishida^1^, Tadashi Imafuku^1,2^, Yuka Nakamura^1^, Hitoshi Maeda^1^, Ayumi Mukunoki^3^, Toru Takeo^3^, Naomi Nakagata^3^, Yuki Kurauchi^4^, Hiroshi Katsuki^4^, Motoko Tanaka^5^, Kazutaka Matsushita^5^, Masafumi Fukagawa^6^, Toru Maruyama^1^

^1^Department of Biopharmaceutics, Graduate School of Pharmaceutical Sciences, Kumamoto University, Kumamoto, Japan; ^2^Program for Leading Graduate Schools "HIGO (Health life science: Interdisciplinary and Glocal Oriented) Program", Kumamoto University, Kumamoto, Japan; ^3^Division of Reproductive Engineering, Center for Animal Resources and Development (CARD), Kumamoto University, Kumamoto, Japan; ^4^Department of Chemico-Pharmacological Sciences, Graduate School of Pharmaceutical Sciences, Kumamoto University, Kumamoto, Japan; ^5^Department of Nephrology, Akebono Clinic, Kumamoto, Japan.; ^6^Division of Nephrology, Endocrinology and Metabolism, Tokai University School of Medicine, Kanagawa, Japan.

*equal contribution: HW, JB and RM

**Supplemental Information**


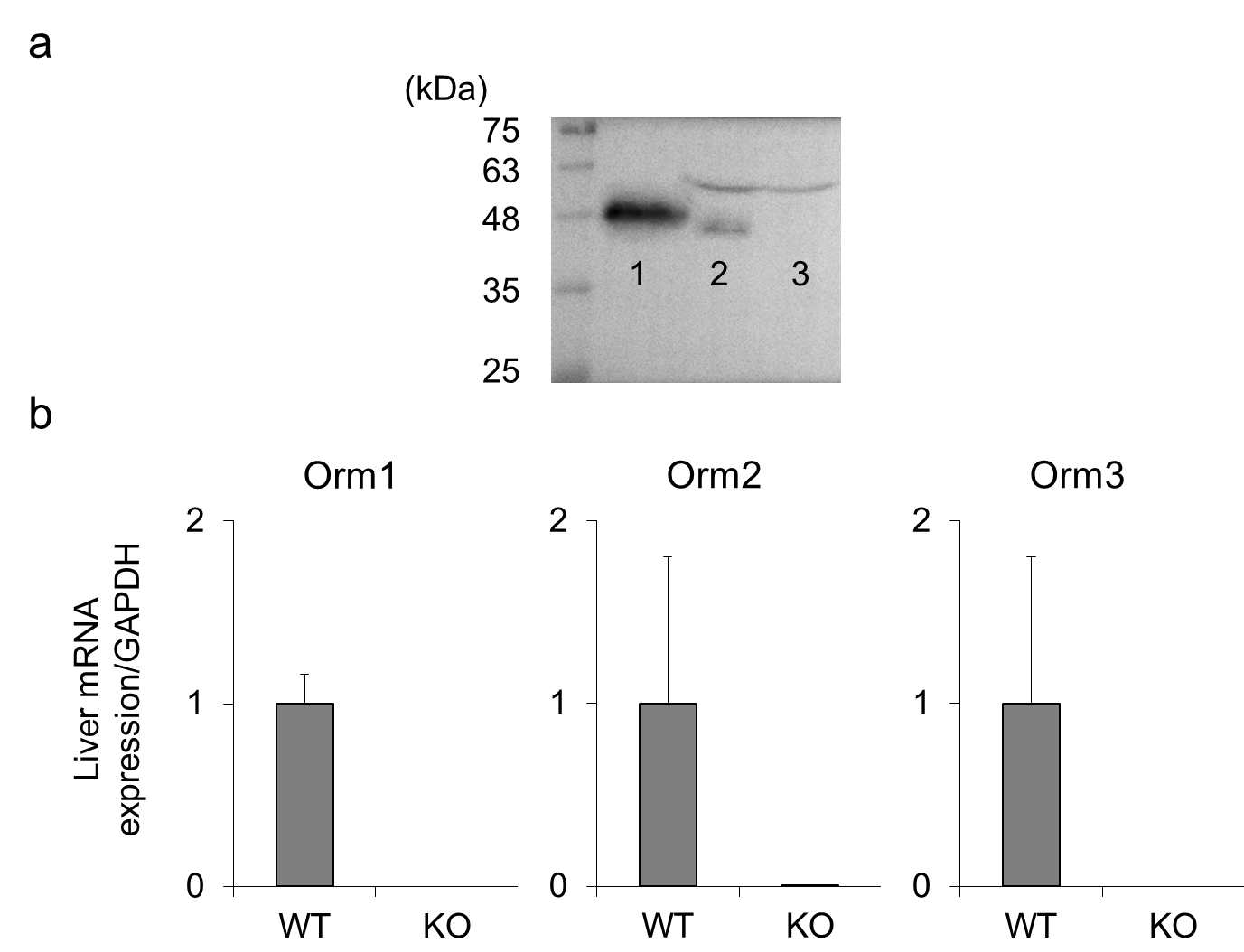


**Supplemental Figure 1.** Confirmation of generating AGP-KO mice.

(a) AGP is not detected in the plasma from AGP-KO mice. Western blot images of AGP derived from human plasma (positive control: lane 1), WT mouse (lane 2) and AGP KO mouse (lane 3).

(b) Liver expression of mRNA of AGP genes, Orm1, Orm2 and Orm3, are not detected in AGP-KO mice.


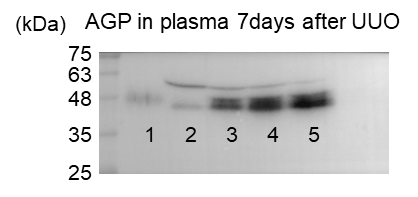


**Supplemental Fig. 2.** Western blot images before cropping into result figures.

Lane 1; AGP derived human plasma (positive control). Lane 2; control mouse. Lane 3; UUO mouse treated with corn oil. Lane 4; UUO mouse treated with atRA. Lane 5; UUO mouse treated with Am80.

Supplemental Table 1. Primers used in real-time RT-PCR

| Target gene | Forward | Reverse |
| --- | --- | --- |
| GAPDH | AACTTTGGCATTGTGGAAGG | ACACATTGGGGGTAGGAACA |
| α−SMA | AGCCATCTTTCATTGGGATGG | CCCCTGACAGGACGTTGTTA |
| Col1a2 | CACCCCAGCGAAGAACTCATA | GCCACCATTGATAGTCTCTCCTAAC |
| IL-1β | TGAGCTGAAAGCTCTCCACC | CTGATGTACCAGTTGGGGAA |
| F4/80 | CATAAGCTGGGCAAGTGGTA | GGATGTACAGATGGGGGATG |

**Supplemental Table 2.** PCR composition, condition, primer sequence and primer


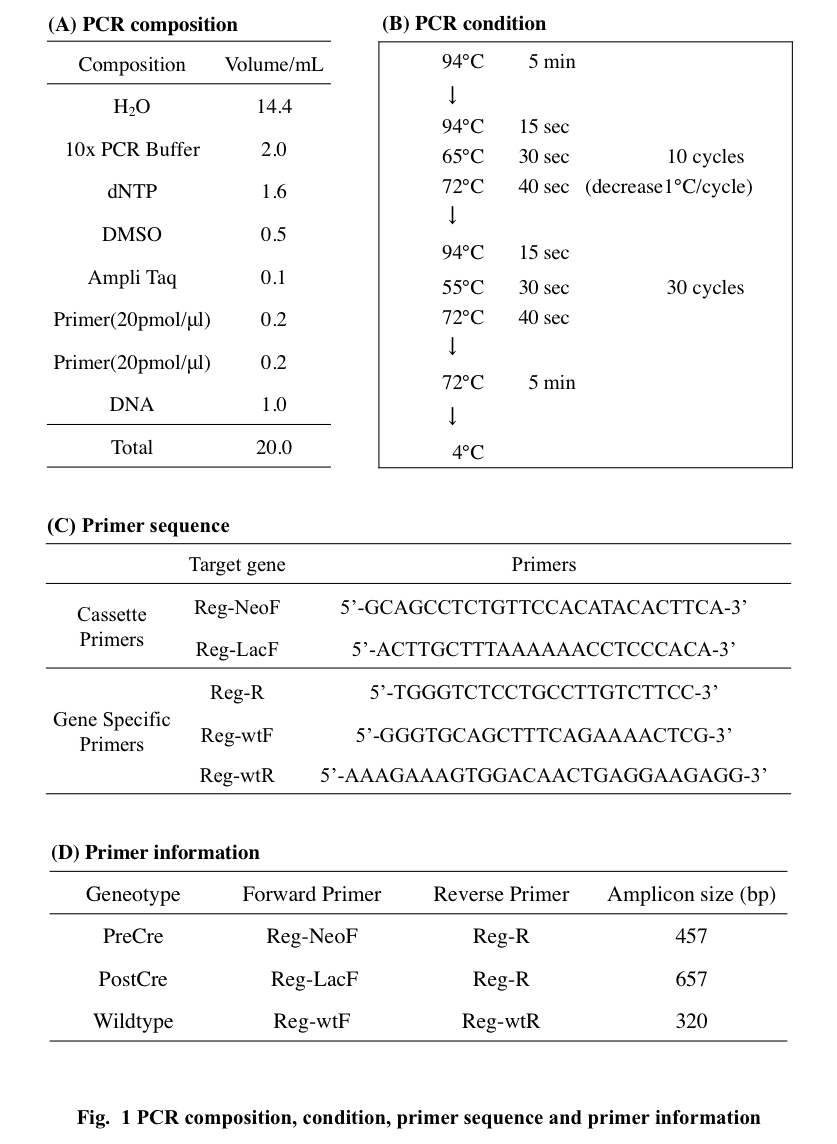


**Expanded Material and Methods:**

**PCR for genotyping**

The last 5 mm of the tail was obtained from the mouse. The tail tissue was then cut into several pieces and placed directly into 300 μL of NaOH (50 mM). Samples heated at 95 °C for 20 min to 1 hr. After heating, the samples were cooled to room temperature and spun down. After adding 30 μL of Tris-HCl (1 M, pH=8) to each sample, it was centrifuged at 13,000 rpm for 5 min. A 1 μL aliquot of the sample was used per each PCR reaction. After DNA isolation by NaOH, the DNA was amplified by PCR. Information regarding the reaction, including PCR composition, PCR condition, primer sequence and primer information is shown in Supplemental Table 2. A touchdown cycling temperature protocol was performed. The 10 x PCR buffer and Ampli Taq were purchased from Applied Biosystems (MA). DNTP was purchased from Takara Bio (Shiga, Japan). Dimethyl sulfoxide (DMSO) was purchased from Nacalai Tesque (Tokyo, Japan). All liquids were prepared as a mixture before applying on the PCR.

**Agarose gel electrophoresis**

To analyze PCR products, a 1 μL aliquot of each PCR product was applied to agarose gel electrophoresis in 2% agarose gel and Tris-Borate-EDTA (TBE) buffer for 50 min. Ethidium bromide (EtBr) was used as a stain and the gel was observed under ultraviolet light.

**Western blotting**

We evaluated mouse AGP in plasm by western blotting following the previously reports^1^. A 0.5 μL aliquot of mouse plasma was separated by 12.5% SDS-PAGE. Proteins were transferred to a PVDF　membrane and then incubated with anti-mouse AGP antibody (1:4000, Proteintech, IL) at room temperature for 1 hr. Each band was detected by LAS 4000mini (GE Healthcare, UK Ltd, Buckinghamshire, England) and quantified using ImageJ software.

Reference

1. Enoki, Y. *et al.* Indoxyl sulfate potentiates skeletal muscle atrophy by inducing the oxidative stress-mediated expression of myostatin and atrogin-1. *Sci Rep* **6**, 32084, doi:10.1038/srep32084 (2016).
